# Surgical Management of a Ruptured Giant Left Main Coronary Artery Aneurysm Presenting with Cardiac Tamponade

**DOI:** 10.3390/diagnostics15182302

**Published:** 2025-09-10

**Authors:** Dmitriy Shumakov, Dmitriy Zybin, Elena Stepanova, Siarhei Dabravolski, Elena Sigaleva, Ekaterina Silina, Victor Stupin, Mikhail Popov

**Affiliations:** 1 M.F. Vladimirsky Moscow Regional Clinical Research Institute, Schepkina St. 61/2, 129110 Moscow, Russia; sdvtranspl@rambler.ru (D.S.); poison1983@inbox.ru (D.Z.); stepanovamoniki@gmail.com (E.S.); popovcardio88@mail.ru (M.P.); 2 Department of Biotechnology Engineering, Braude Academic College of Engineering, Snunit 51, P.O. Box 78, Karmiel 2161002, Israel; 3 Institute of Biomedical Problems, Russian Academy of Sciences, 76A Khoroshevskoye Shosse, 123007 Moscow, Russia; sigaleva@mail.ru; 4 Department of Hospital Surgery, Pirogov Russian National Research Medical University, 117997 Moscow, Russia; silinaekaterina@mail.ru (E.S.); stvictor@bk.ru (V.S.)

**Keywords:** giant coronary artery aneurysm, coronary artery rupture, cardiac tamponade, coronary artery bypass grafting

## Abstract

Coronary artery aneurysms (CAAs) are an uncommon finding, and their rupture is an exceedingly rare and life-threatening complication. Giant aneurysms of the left main coronary artery (LMCA) pose a significant diagnostic and therapeutic challenge. We describe the case of a 62-year-old male who presented with acute coronary syndrome and was subsequently diagnosed with a ruptured giant LMCA aneurysm causing cardiac tamponade and multi-organ dysfunction. The initial diagnosis was suggested by coronary angiography and confirmed with contrast-enhanced multidetector computed tomography (MDCT) and echocardiography. The patient underwent emergency surgery consisting of aneurysm excision, thrombectomy, ligation of the LMCA ostium and its distal branches (LAD and circumflex), and coronary artery bypass grafting (CABG) using the left internal thoracic artery to the left anterior descending artery and a saphenous vein graft to a marginal branch. The patient’s postoperative course was complicated by transient multi-organ dysfunction, which resolved. He was discharged in a stable condition. This case highlights the critical importance of rapid multimodal imaging for diagnosis and the feasibility of emergency surgical intervention to achieve a favorable outcome in patients with a ruptured giant LMCA aneurysm.

**Figure 1:**
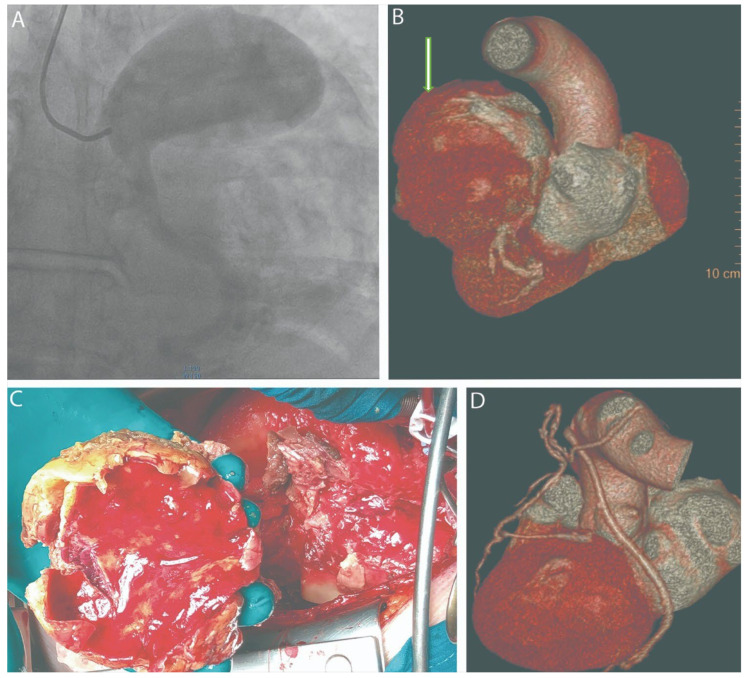
(**A**) Coronary angiography; (**B**) MDCT before surgery (the white arrow indicates an aneurysm of the trunk of the left coronary artery); (**C**) thrombotic masses in aneurysmal sac; (**D**) MDCT after surgery. A coronary artery aneurysm (CAA) is defined as a localized dilation of a coronary artery exceeding the diameter of an adjacent normal segment by 1.5 times. CAAs are identified in approximately 1.2–4.9% of patients undergoing coronary angiography and in 1.4% of autopsy series, with a higher prevalence in males [1]. While vasculitis, particularly Kawasaki disease, is a primary etiology in pediatric populations [2], atherosclerosis is the most common cause in adults. Although often asymptomatic, these lesions are not benign; they can lead to severe complications such as thrombosis and distal embolization, and, if left untreated, can be associated with significant cardiomyopathy and fatal outcomes, as highlighted in autopsy reports [3]. Aneurysms with a diameter exceeding 4 cm are classified as “giant” aneurysms [4]. A ruptured giant aneurysm of the left main coronary artery (LMCA) is an exceptionally rare event with catastrophic hemodynamic consequences [5,6]. Herein, we report a case of a ruptured giant LMCA aneurysm presenting with hemopericardium and cardiac tamponade that was successfully managed with emergency surgery. A 62-year-old male was admitted to a regional hospital with symptoms of exertional angina, diagnosed as an ST-elevation acute coronary syndrome. An emergent coronary angiography revealed a giant aneurysm of the LMCA (**A**). Concurrently, transthoracic echocardiography demonstrated a large pericardial effusion consistent with hemopericardium and signs of cardiac tamponade. Due to his critical condition, the patient was urgently transferred to our tertiary care center. A contrast-enhanced multidetector computed tomography (MDCT) scan confirmed the presence of a giant, partially thrombosed LMCA aneurysm measuring 13 cm × 8 cm, with evidence of rupture and associated hemopericardium (**B**). The patient was taken for emergency surgery. In anticipation of potential hemodynamic collapse upon sternotomy, the femoral artery and vein were prepared for emergent cannulation. Following a median sternotomy, the pericardium was opened, and approximately 800 mL of lysed blood was evacuated, resulting in immediate hemodynamic stabilization. Cardiopulmonary bypass (CPB) was initiated via aortic and right atrial cannulation. After achieving cardioplegic arrest, with an antegrade infusion of 2000 mL of cold Custodiol^®^ solution into the aortic root, the aneurysmal sac was incised at the site of the suspected rupture. The lumen contained extensive thrombotic masses of varying ages, which were completely evacuated. The resected aneurysmal wall was sent for histopathological examination, which subsequently revealed findings of connective tissue dysplasia and cystic medial necrosis (**C**). The ostia of the LMCA, left anterior descending (LAD) artery, and circumflex artery were identified from within the aneurysm and securely ligated. Coronary artery bypass grafting (CABG) was then performed. The LAD was revascularized with the in situ left internal thoracic artery (LITA), and an obtuse marginal branch was bypassed using a saphenous vein graft. The redundant walls of the aneurysmal sac were resected. The remaining cavity was closed with a two-row continuous suture, reinforced with xenopericardial strips to ensure hemostasis. The patient was weaned from CPB with minimal inotropic support, and the chest was closed in a standard fashion. The early postoperative period was complicated by transient multi-organ dysfunction, which was managed with supportive care in the intensive care unit (ICU) and resolved by postoperative day 5. A multispiral computed tomography of the heart was performed after surgery with 3D reconstruction (**D**). The patient was subsequently transferred to the cardiac surgery ward and was discharged home on postoperative day 14 in a satisfactory clinical condition. The etiology of giant coronary aneurysms in adults is most commonly atherosclerotic. However, the histopathological findings in our case, which revealed connective tissue dysplasia and cystic medial necrosis, point towards a non-atherosclerotic, underlying structural defect of the vessel wall. This finding is significant as it underscores that other degenerative processes must be considered in the differential diagnosis, even in older patients. A coronary artery aneurysm is defined as a coronary artery dilation exceeding 1.5 times the diameter of a contiguous normal segment, with those larger than 4 cm classified as giant [7]. While the diagnostic incidence of CAAs has increased with the widespread use of coronary angiography and CT angiography, reports of giant aneurysms of the LMCA remain rare, and cases complicated by rupture are exceptional [8]. The management of giant CAAs is not standardized due to their low incidence [9]. However, the presence of symptoms or complications such as rupture, as seen in our patient, is an absolute indication for open-heart surgery. This aligns with expert consensus, which advises surgical treatment for giant aneurysms, especially those involving the LMCA or presenting with mechanical complications like rupture [10]. A ruptured aneurysm creates a surgical emergency due to the rapid development of cardiac tamponade and subsequent hemodynamic collapse. The diagnostic pathway in our case, utilizing initial coronary angiography followed by MDCT, proved critical for preoperative planning. While conventional angiography can identify an aneurysm, MDCT offers superior spatial resolution for defining the precise anatomy, extent of thrombosis, and relationship to adjacent cardiac structures. The value of MDCT in delineating complex coronary anomalies for therapeutic planning has been well-documented, making it an essential adjunct to angiography [11]. Our surgical strategy prioritized immediate hemodynamic stabilization by relieving the tamponade, followed by definitive treatment on CPB. The chosen technique—aneurysmectomy with ligation of inflow and outflow vessels combined with distal revascularization via CABG—is a well-established approach for such complex cases. This strategy has been shown to be effective in other case series of giant CAAs, where aneurysm exclusion and bypass grafting led to excellent outcomes [12]. Furthermore, in acute presentations such as cardiogenic shock, emergent CABG is the preferred approach, with ligation of the aneurysm strongly recommended in patients with thromboembolic phenomena [13]. The extensive thrombotic burden in our patient’s aneurysm made this combined approach of ligation and bypass imperative to prevent future ischemic or embolic events. The intra-aneurysmal ligation of the LMCA, LAD, and circumflex ostia is a critical step to prevent recurrent bleeding and “steal” phenomenon. This report is limited by its nature as a single case study. Therefore, the outcomes and management strategy described cannot be generalized. Long-term follow-up will be necessary to assess the durability of the repair and the patency of the bypass grafts. Ruptured giant LMCA aneurysms are a rare but lethal condition. This case demonstrates that with prompt and accurate diagnosis using a multimodal imaging approach (angiography, echocardiography, and CT), followed by emergent surgical intervention, a favorable outcome is achievable despite the presence of life-threatening complications like cardiac tamponade and multi-organ dysfunction.

## Data Availability

The original contributions presented in this study are included in the article. Further inquiries can be directed to the corresponding author due to ethical reasons.
